# Metformin attenuates gefitinib-induced exacerbation of pulmonary fibrosis by inhibition of TGF-β signaling pathway

**DOI:** 10.18632/oncotarget.6186

**Published:** 2015-10-20

**Authors:** Li Li, Wenting Huang, Kunlin Li, Kejun Zhang, Caiyu Lin, Rui Han, Conghua Lu, Yubo Wang, Hengyi Chen, Fenfen Sun, Yong He

**Affiliations:** ^1^ Department of Respiratory Disease, Daping Hospital, Third Military Medical University, Chongqing, China; ^2^ Department of Clinical Labratory, Daping Hospital, Third Military Medical University, Chongqing, China

**Keywords:** metformin, EGFR-TKI, pulmonary fibrosis, TGF-β

## Abstract

Interstitial lung disease (ILD) is a serious side-effect of epidermal growth factor receptor (EGFR)-tyrosine kinase inhibitor (TKI) treatment. Therefore, it is necessary to study underlying mechanisms for the development of pulmonary fibrosis induced by EGFR-TKI and potential approaches to attenuate it. Metformin is a well-established and widely prescribed oral hypoglycemic drug, and has gained attention for its potential anticancer effects. Recent reports have also demonstrated its role in inhibiting epithelial-mesenchymal transition and fibrosis. However, it is unknown whether metformin attenuates EGFR-TKI-induced pulmonary fibrosis. The effect of metformin on EGFR-TKI-induced exacerbation of pulmonary fibrosis was examined *in vitro* and *in vivo* using MTT, Ki67 incorporation assay, flow cytometry, immunostaining, Western blot analysis, and a bleomycin-induced pulmonary fibrosis rat model. We found that in lung HFL-1 fibroblast cells, TGF-β or conditioned medium from TKI-treated lung cancer PC-9 cells or conditioned medium from TKI-resistant PC-9GR cells, induced significant fibrosis, as shown by increased expression of Collegen1a1 and α-actin, while metformin inhibited expression of fibrosis markers. Moreover, metformin decreased activation of TGF-β signaling as shown by decreased expression of pSMAD2 and pSMAD3. *In vivo*, oral administration of gefitinib exacerbated bleomycin-induced pulmonary fibrosis in rats, as demonstrated by HE staining and Masson staining. Significantly, oral co-administration of metformin suppressed exacerbation of bleomycin-induced pulmonary fibrosis by gefitinib. We have shown that metformin attenuates gefitinib-induced exacerbation of TGF-β or bleomycin-induced pulmonary fibrosis. These observations indicate metformin may be combined with EGFR-TKI to treat NSCLC patients.

## INTRODUCTION

Reversible small-molecule epidermal growth factor receptor (EGFR)-tyrosine kinase inhibitors (TKIs), including gefitinib (Iressa) and erlotinib (Tarceva), display dramatic therapeutic efficacy in NSCLC patients that have EGFR-activating mutations, and are recommended as the standard first-line therapy in NSCLC [1, 2]. However, despite excellent initial clinical responses, these drugs might promote interstitial lung disease (ILD), which is a less common but lethal side-effect that restricts the therapeutic efficacy of these agents [3]. The incidence of EGFR-TKI-associated ILD has been described for both gefitinib (2.4%) and erlotinib (1%) [4]. In addition, the incidence of EGFR-TKI-associated ILD appears comparatively higher in Asians than in Caucasians, as demonstrated in two large, multi-institutional studies reporting its incidence in Japan at 3.5–4.0% [5, 6], as compared with that of 0.3% in the U.S. [7]. No specific treatment is available for EGFR-TKI-associated ILD, and supportive therapy largely includes oxygen, corticosteroids, or assisted ventilation, with an approximate 30%–40% mortality of the disease. Thus, acute and innovative treatment strategies are urgently needed to overcome the lethal side-effect of EGFR-TKIs.

Common presentations of ILD includes pulmonary fibrosis and pneumonia. Molecular mechanisms underlying TKI-induced interstitial pneumonia (i.e., pulmonary fibrosis) remain incompletely understood. A key mediator in the pathogenesis of pulmonary fibrosis is transforming growth factor (TGF)-β1 [8]. TGF-β1 is a member of the TGF-β superfamily, and is a pleiotropic cytokine that is ubiquitously expressed by all cells and tissues that regulates a range of biological functions from embryonic development to wound repair, and does so primarily through a canonical Smad-dependent pathway [9]. High expression of TGF-β1 is found in tissue samples from both animal models of IPF [10] and patients with IPF [11]. Over-expressing active TGF-β1 results in persisting pulmonary fibrosis [12], whereas blocking TGF-β signaling ameliorates pulmonary fibrosis in animal models [13, 14].

The progressive pulmonary fibrotic reaction was also associated with an epithelial-dependent fibroblast-activating process, which is termed epithelial–mesenchymal transition (EMT) [15, 16]. TGF-β was shown to be a master inducer of EMT in normal mammary epithelial cells and was further proven to initiate and maintain EMT in the organ fibrogenic and tumor metastasis model [17]. In the lung, repeated acute injury provokes cell death and increases the proliferative capacity and abnormal activation of alveolar epithelial cell (AECs), which secrete latent TGF-β1. Collectively, this promotes alveolar EMT in AECs and transdifferentiation of quiescent fibroblasts into myofibroblasts, which contribute to excessive production of fibrillar collagens [18]. Importantly, IL-6, which is a downstream molecule of TGF-β activation, is suggested to contribute to the development or progression of acute interstitial pneumonia [19, 20]. EGFR-TKI treatment increased IL-6 secretion in cancer cells, which further induces acute interstitial pneumonia [21]. Thus, given the established actions of TGF-β on EMT and collagen synthesis, pharmacological inhibition of TGF-β signaling might exhibit important therapeutic potential in the clinical treatment of pulmonary fibrosis.

Up to now, no substantial therapeutic interventions have been developed to reverse established fibrosis or even halt chronic progression to respiratory failure. One of the most widely used oral agents to lower blood glucose levels in patients with type 2 diabetes and metabolic syndrome is the biguanide derivative metformin. Earlier studies by us revealed that clinically-relevant doses of metformin notably reversed EGFR-TKI resistance by inhibiting the IL-6 signaling pathway and reversing EMT [22]. This observation prompted us to investigate whether metformin could attenuate TKI-induced pulmonary fibrosis. Indeed, studies have shown that metformin may play a role in preventing fibrosis [23]. However, it is unknown whether metformin attenuates EGFR-TKI-induced pulmonary fibrosis.

Herein, we show that metformin effectively decreased gefitinib-induced fibrosis in lung fibroblast HFL-1 cells. In addition, metformin attenuates gefitinib-exacerbated pulmonary fibrosis in a rat model of lung injury that was induced by bleomycin. This effect was associated with metformin-mediated decreases in the activation of the TGF-β/IL-6 signaling pathway and reversal of EMT. We thus provide a rationale and experimental evidence for using metformin to attenuate pulmonary fibrosis that is induced by EGFR-TKIs.

## RESULTS

### Metformin decreased TGF-β-induced pulmonary fibrosis *in vitro*

**Figure 1:**
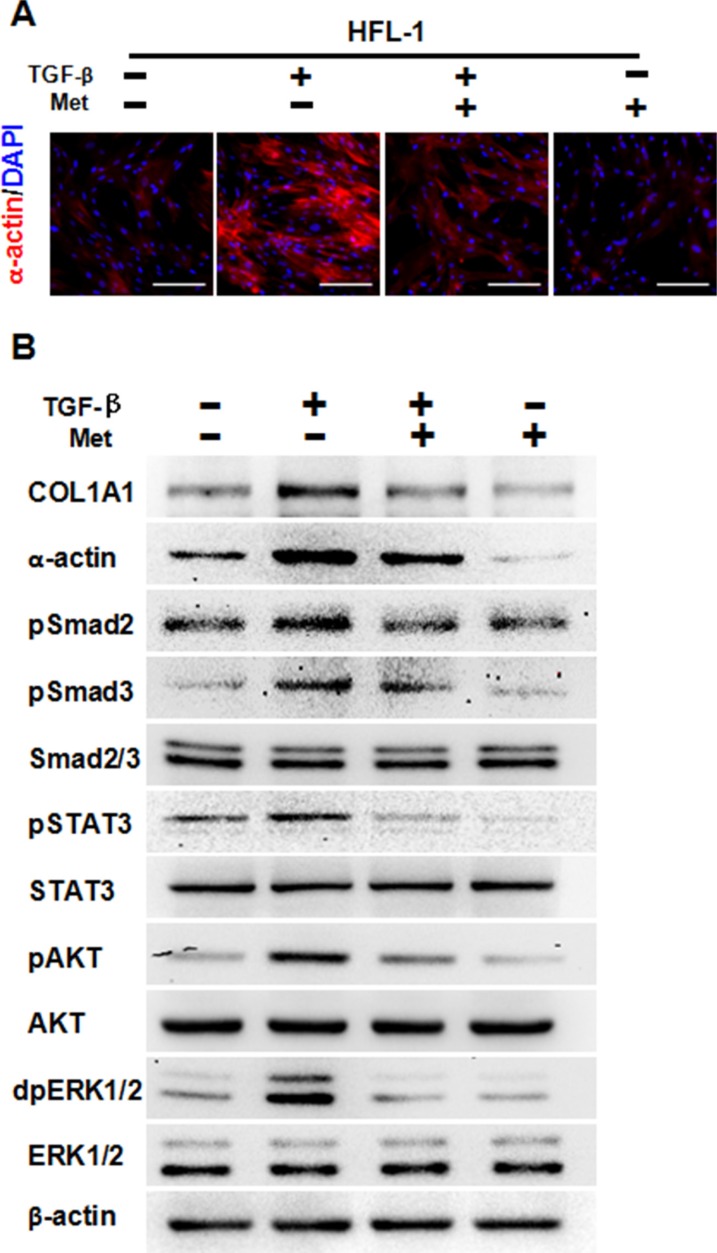
Metformin attenuates TGF-β-induced fibrosis **(A)** Immunofluorescence staining showed that metformin decreased TGF-β-induced expression of α-actin in HFL-1 cells. The nucleus were stained with 4′, 6-diamidino-2-phenylindole in the merged images. Scale bars: 150 μm. **(B)** Metformin inhibited TGF-β-induced fibrosis as well as downstream signaling pathways, as determined by western blot analysis. Whole cell protein lysates from HFL-1 cells of different treatments were immunoblotted with indicated antibodies. Similar results were obtained in three independent experiments. Met, metformin.

We first aimed to determine whether metformin could decrease TGF-β-induced pulmonary fibrosis. In lung fibroblast HFL-1 cells, treatment with 10 ng/ml TGF-β for 48 h induced expression of α-actin, a marker of fibrosis, as demonstrated by immunofluorescence staining (Figure 1A). Of note, addition of 5 mM metformin decreased expression levels of α-actin, while metformin alone also decreased α-actin expression (Figure 1A). Western blot results further confirmed that metformin decreased TGF-β-induced expression of α-actin and Collagen 1A1 (COL1A1), another marker of fibrosis (Figure 1B). Furthermore, TGF-β induced the activation of pSMAD2, pSMAD3, pSTAT3, pAKT and dpERK1/2, while addition of metformin decreased expression levels of those phosphorylated proteins (Figure 1B). Taken together, these data suggest that metformin decreased TGF-β-induced pulmonary fibrosis *in vitro*.

### Metformin decreased pulmonary fibrosis induced by conditioned medium from EGFR-TKI-treated lung cancer cells and EGFR-TKI resistant human lung cancer cells *in vitro*

**Figure 2:**
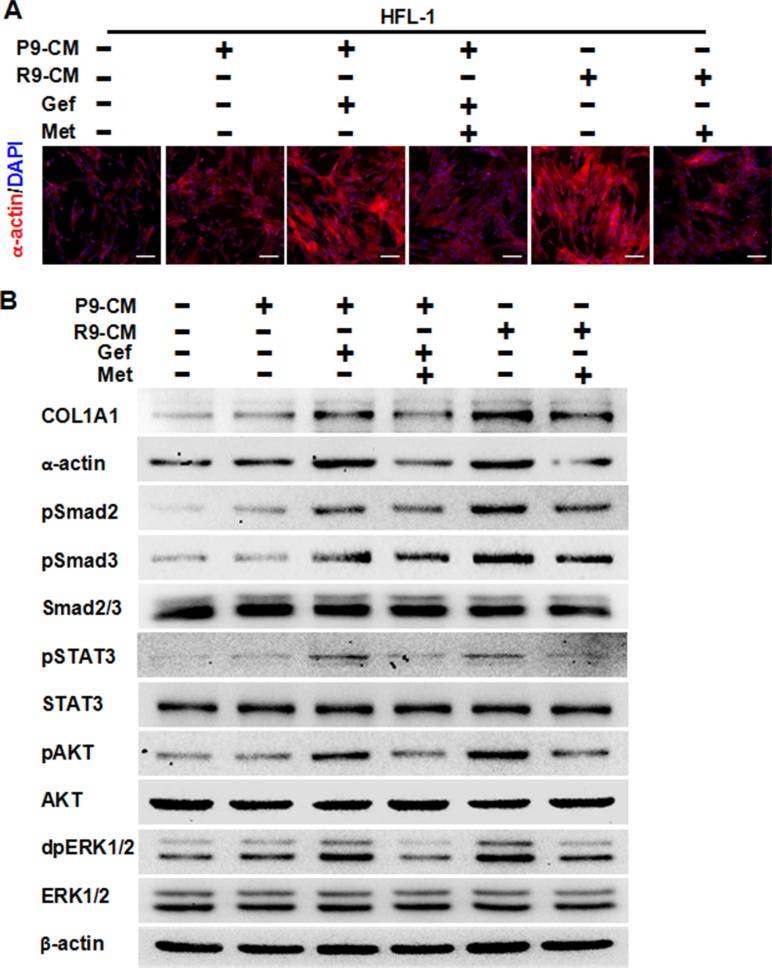
Metformin attenuates fibrosis induced by conditioned medium from TKI-treated lung cancer PC-9 cells or conditioned medium from TKI-resistant PC-9GR cells **(A)** Immunofluorescence staining showed that metformin decreased expression of α-actin in HFL-1 cells either induced by conditioned medium from TKI-treated lung cancer PC-9 cells or conditioned medium from TKI-resistant PC-9GR cells. The nucleus were stained with 4′, 6-diamidino-2-phenylindole in the merged images. Scale bars: 150 μm. **(B)** Metformin decreased TKI-induced expression of COL1A1 and α-actin, and inhibited TKI-enhanced expression of pSMAD2, pSMAD3, pSTAT3, pAKT, and dpERK1/2, as shown by western blot assay. Whole cell protein lysates from HFL-1 cells with different treatments were immunoblotted with antibodies as indicated, and β-actin was used to confirm equal gel loading. Similar results were obtained in three independent experiments. P9, PC-9 cells; R9, PC-9GR cells; CM, conditioned medium; Gef, gefitinib; Met, metformin.

Next, we studied whether TKI treatment induced pulmonary fibrosis and whether metformin could decrease it. To this end, we used two cell-lines, the parental TKI-sensitive PC-9 cells and TKI-resistant PC-9GR cells. We treated HFL-1 cells with conditioned medium from PC-9 cells, gefitinib-treated PC-9 cells or PC-9GR cells, and examined the fibrotic condition by both immunostaining and Western blot assay. As expected, conditioned medium from PC-9 cells did not induce fibrosis, since the expression of α-actin and COL1A1 were quite similar to that of the control. Conditioned medium from either gefitinib-stimulated PC-9 cells or from PC-9GR cells induced high expression of both α-actin and COL1A1. Importantly, the addition of 5 mM metformin significantly decreased expression of both markers of fibrosis (Figure 2A and 2B). Furthermore, metformin inhibited gefitinib-induced over-expression of pSMAD2, pSMAD3, pSTAT3, pAKT and dpERK1/2. Based on these findings, we concluded that metformin decreased EGFR-TKI-induced pulmonary fibrosis *in vitro*.

### TGF-β was required for EGFR-TKI-treated lung cancer cells and EGFR-TKI resistant human lung cancer cells to induce pulmonary fibrosis

**Figure 3:**
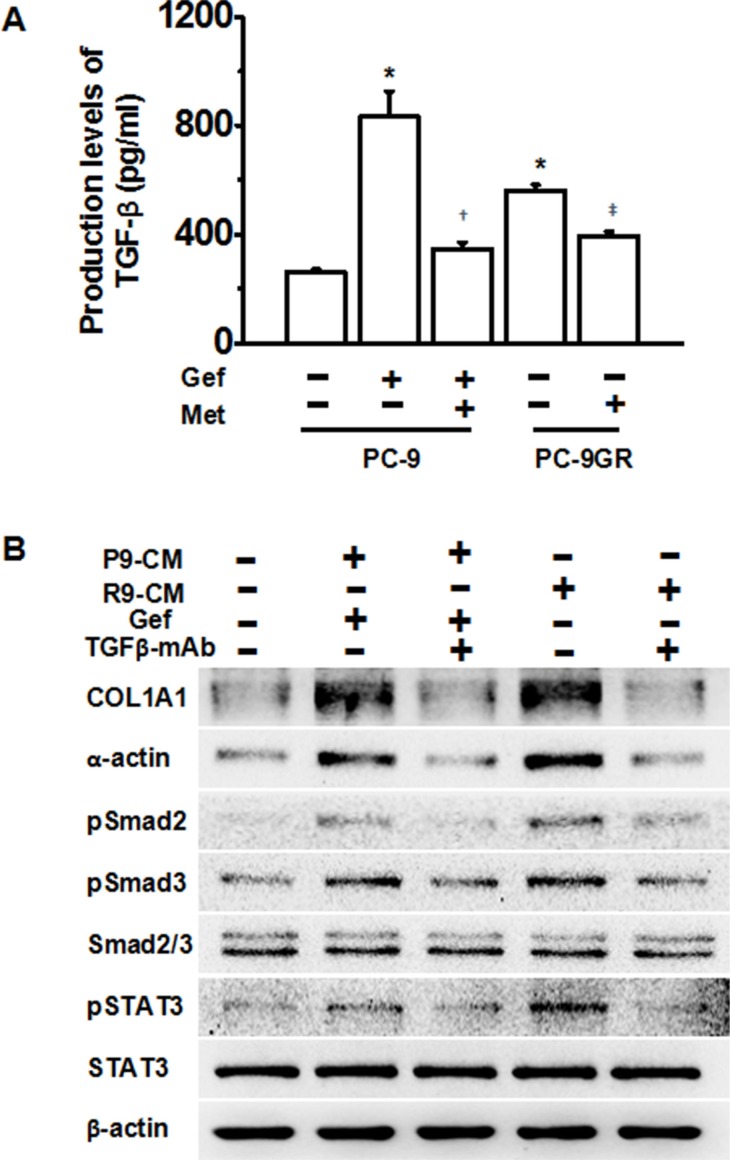
Metformin attenuates TKI-induced fibrosis through down-regulation of TGF-β signaling pathway **(A)** Metformin significantly decreased TGF-β secretion levels in TKI-treated PC-9 cells and PC-9GR cells as determined by ELISA assays. *p < 0.01 compared with PC-9 cells of no treatment, respectively; ^†^*p* < 0.01, compared with PC-9 cells with gefitinib treatment; ^‡^*p* < 0.05, compared with untreated PC-9GR cells. Met, metformin; Gef, gefitinib. **(B)** Addition of TGF-β mAb abolished TKI-induced fibrosis. HFL-1 cells were treated as indicated and whole cell protein lysates were immunoblotted with antibodies as indicated, and β-actin was used to confirm equal gel loading. Similar results were obtained in three independent experiments. P9, PC-9 cells; R9, PC-9GR cells; CM, conditioned medium; Gef, gefitinib.

We next asked whether metformin decreased EGFR-TKI-induced pulmonary fibrosis by inhibiting the TGF-β signaling pathway. We first performed ELISA assay, and found higher levels of protein secretion of TGF-β in gefitinib-treated PC-9 cells and gefitinib-resistant PC-9GR cells. Metformin treatment significantly decreased TGF-β protein secretion in both cell-lines (Figure 3A). We next asked whether TGF-β was required for gefitinib treatment-induced pulmonary fibrosis. Treatment with conditioned medium from gefitinib-stimulated PC-9 cells or PC-9GR cells induced high expression of COL1A1 and α-actin, while addition of a TGF-β monoclonal antibody (mAb) abolished the pro-fibrotic effects of both conditioned medium. Also, TGF-β mAb decreased activation of pSMAD2, pSMAD3 and pSTAT3. Thus, we conclude that the TGF-β pathway plays a central role in gefitinib-induced fibrosis, and metformin decreased gefitinib-induced fibrosis by down-regulating the TGF-β pathway.

### Metformin decreases TGF-β-induced fibrosis depending on AMPK activation

**Figure 4:**
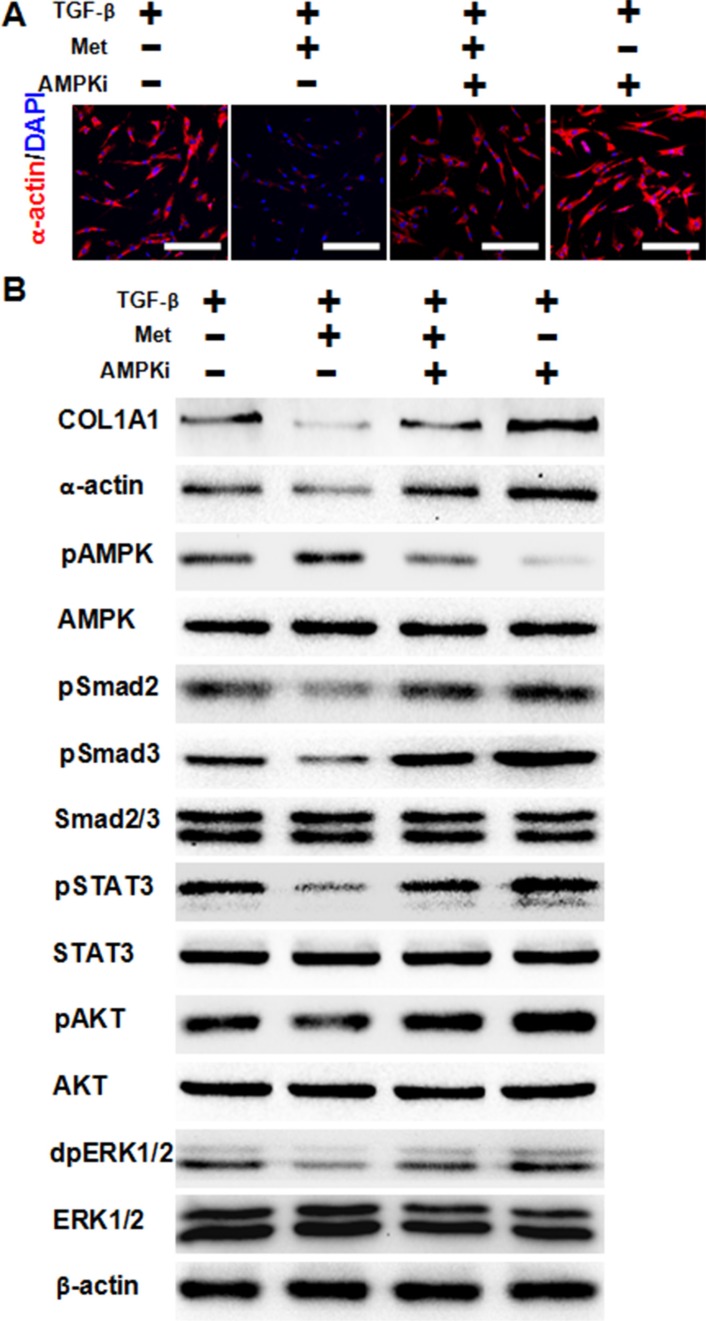
AMPK activation is required for metformin to attenuate TGF-β-induced fibrosis **(A)** Cells were pre-treated with 10 ng/ml TGF-β for 24 h, then 5 mM metformin and/or 1 uM (μM) dorsomorphin were added to the medium for another 24 h. Immunofluorescence staining showed that AMPK inhibition abolished metformin's effect of decreasing TGF-β-induced α-actin expression in HFL-1 cells. The nucleus were stained with 4′, 6-diamidino-2-phenylindole in the merged images. Scale bars: 150 μm. **(B)** AMPK inhibition abolished metformin's effect of decreasing TGF-β signaling pathways, as determined by western blot analysis. Whole cell protein lysates from HFL-1 cells of different treatments were immunoblotted with indicated antibodies. Similar results were obtained in three independent experiments. Met, metformin; AMPKi, AMPK inhibition.

Previous studies have shown that activation of AMPK by metformin prevents TGF-β-induced EMT and attenuates tubulointerstitial fibrosis. Thus, we next ask whether AMPK activation was required for metformin to decrease TGF-β-induced fibrosis in HFL-1 cells. As showen in Figure 4A, immunofluorescence staining showed that metformin addition decreased expression of α-actin induced by TGF-β, while further addition of an AMPK inhibitor abolished this effect. Furthermore, the use of an AMPK inhibitor abolished the effect of metformin in decreasing the activation of downstream signaling pathways of TGF-β (Figure 4B). These data suggest that activation of AMPK by metformin was required to decrease TGF-β-induced fibrosis.

### Metformin inhibits cell proliferation and induces progressive apoptosis in HFL-1 cells

**Figure 5:**
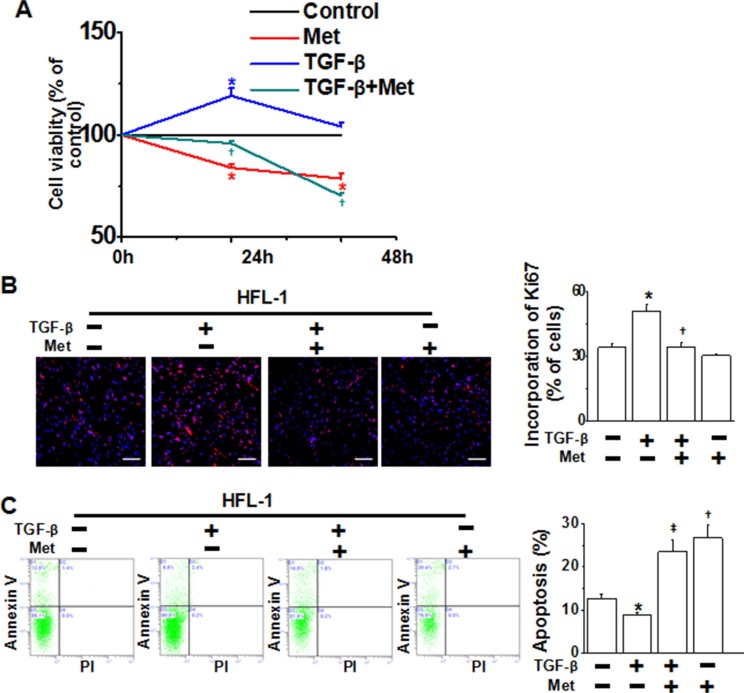
Metformin inhibits cell proliferation and promotes apoptosis in HFL-1 cells **(A)** HFL-1 cells were treated as indicated for 24 h and 48 h, respectively. Cell viability, which was assessed by the MTT assay, was expressed as % of control for each time point. *p < 0.05 compared with control of the same time point, ^†^*p* < 0.05 compared with TGF-β alone of the same time point. **(B)** Metformin inhibited TGF-β-induced proliferation of HFL-1 cells, as determined by ki67 incorporation assay. *p < 0.01 compared with control, ^†^*p* < 0.01 compared with TGF-β alone. Scale bars, 150 μm. **(C)** Metformin enhanced apoptosis of HFL-1 cells. Images are representative of three independent experiments. *p < 0.05 compared with control; ^†^*p* < 0.01 compared with control; ^‡^*p* < 0.01 compared with TGF-β treatment.

Since fibroblasts undergo autonomous proliferation and produce excessive matrix proteins that resemble a wound-healing process during pulmonary fibrosis [24], we subsequently investigated the ability of metformin to modulate fibroblast proliferation and apoptosis in HFL-1 cells. As determined by MTT assay, TGF-β stimulation for 24 h increased the number of viable fibroblasts, whereas the cell viability was reduced by metformin (Figure 5A). As metformin disrupts mitochondrial respiration, which may affect the performance and results of the MTT assay, we explored the impact of metformin on cell growth using the Ki67 assay. As shown in Figure 5B, DNA synthesis was rapidly decreased in the cells following treatment with metformin. We next analyzed the induction of apoptosis in HFL-1 cells treated with TGF-β alone or in combination with metformin. Flow cytometric analysis revealed that TGF-β alone inhibited apoptosis of HFL-1 cells, while metformin augmented cell apoptosis (Figure 5C). Taken together, metformin inhibited cell proliferation and induced progressive apoptosis in HFL-1 cells.

### Metformin attenuates EGFR-TKI-induced exacerbation of pulmonary fibrosis *in vivo*

**Figure 6:**
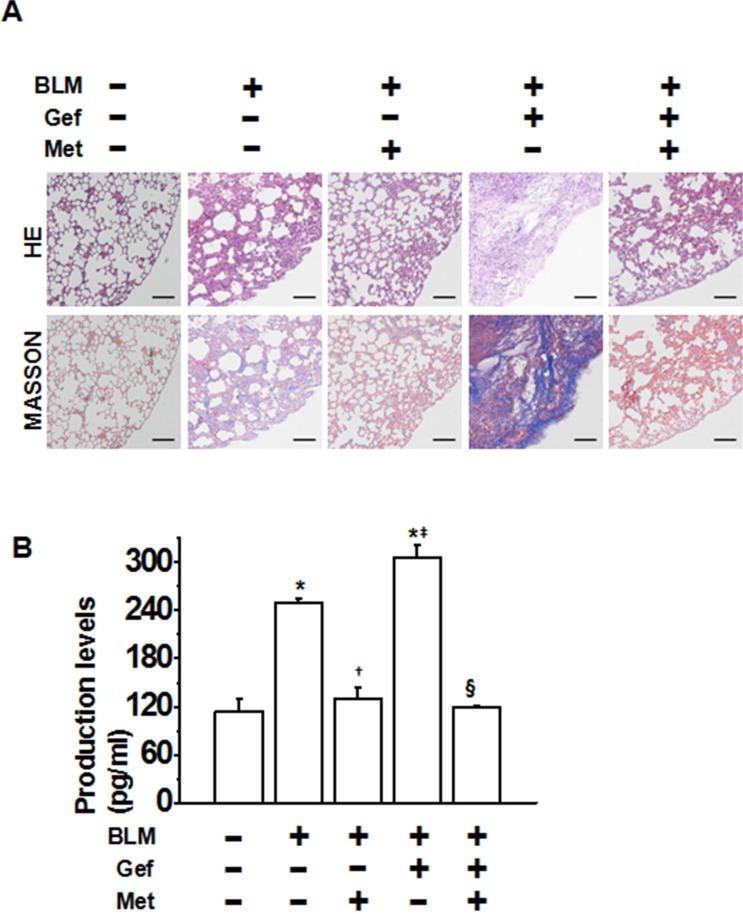
Metformin ameliorates TKI-induced exacerbation of bleomycin-induced pulmonary fibrosis *in vivo* **(A)** Pulmonary tissue sections were prepared at day 21 and subjected to hematoxylin and eosin (H&E) for routine examination and Masson's trichrome staining for the visualization of collagen deposition. Scale bars = 150 μm. **(B)** The hydroxyproline (Hyp) content of the pulmonary tissues (*n* = 5 for each group) was measured at day 28 and is presented as micrograms of Hyp per gram of wet weight (mg/g). *p < 0.01 compared with control; ^†^*p* < 0.05 compared with bleomycin treatment alone; ^‡^*p* < 0.05 compared with bleomycin treatment alone; ^§^*p* < 0.01 compared with bleomycin + gefitinib treatment. BLM, bleomycin; Gef, gefitinib; Met, metformin.

We next tested the possibility of whether metformin attenuates EGFR-TKI-induced exacerbation of pulmonary fibrosis. We first established an acute lung injury model by bleomycin (BLM). As determined by hematoxylin and eosin staining of lung sections, the intratracheal injection of BLM led to the destruction of normal pulmonary architecture, the prominent proliferation of fibroblasts, the infiltration of inflammatory cells and the extensive deposition of fibrillar collagen. Furthermore, oral administration of gefitinib produced more severe pulmonary damage. Impressively, we observed remarkable improvement in these pathological changes after the administration of metformin (Figure 6A). Likewise, the deposition of collagen fibers was largely increased by gefitinib treatment in BLM-induced lung injury. By contrast, it was significantly reduced following administration of metformin, as illustrated by Masson's trichrome-positive staining (Figure 6A). We then measured the pulmonary hydroxyproline (Hyp) content of eight mice from each group to quantify the extent of pulmonary fibrosis, as Hyp is a major constituent of collagen. Both BLM and BLM plus gefitinib induced high levels of Hyp; by contrast, metformin administration significantly decreased Hyp levels. Taken together, these data suggest a protective role of metformin in attenuating EGFR-TKI-induced exacerbation of pulmonary fibrosis *in vivo*.

### Metformin inhibits the TGF-β signaling pathway and the EMT phenotype *in vivo*

**Figure 7:**
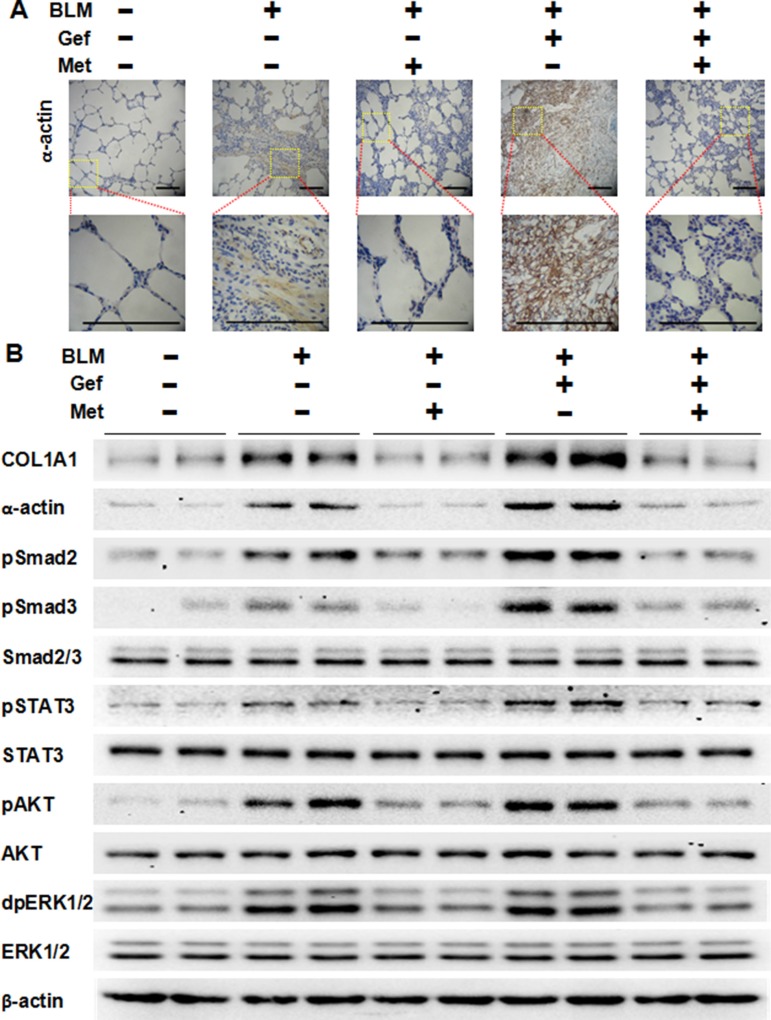
Metformin inhibits TGF-β signaling pathway *in vivo* **(A)** Metformin decreased expression of α-actin in fibrotic lung tissues from different groups treated as indicated. Paraffin-embedded sections (4 μm) from lung tissues were stained for α-actin using immunohistochemistry. The area indicated by the square is shown at higher magnification. Scale bars = 150 μm. **(B)** Western blotting analyzed the expression of indicated markers on protein extracts obtained from lung tissues, and β-actin was used as a loading control. BLM, bleomycin; Gef, gefitinib; Met, metformin.

**Figure 8:**
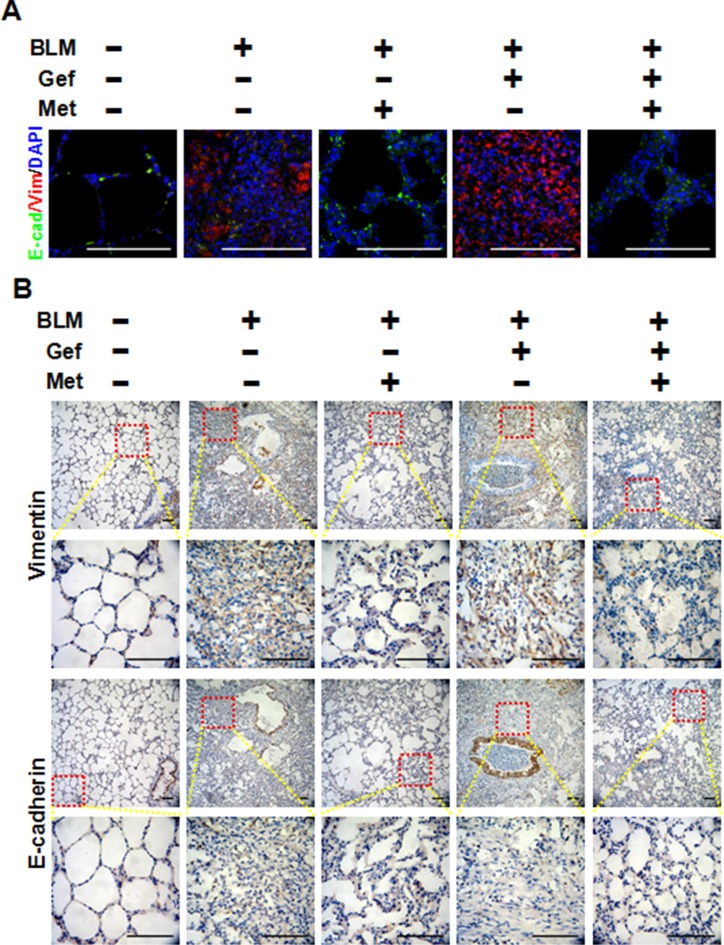
Metformin inhibits the EMT phenotype *in vivo* **(A)** Metformin reversed EMT in fibrotic lung tissue, as shown by immunefluorescence staining. Each image depicts a representative immunostaining of a paraffin-embedded section (4 μm) for E-cadherin in green, Vimentin in red and counter-staining with 4′, 6-diamidino-2-phenylindole (DAPI) in blue. Scale bars = 100 μm. **(B)** Immunohistochemistry was used to examine the expression of E-cadherin and Vimentin in lung tissues from different groups of mice treated as indicated. The area indicated by the square is shown at higher magnification. Scale bars = 150 μm. BLM, bleomycin; Gef, gefitinib; Met, metformin.

To clarify the underlying mechanism of why metformin attenuates EGFR-TKI-induced exacerbation of pulmonary fibrosis *in vivo*, we next analyzed the TGF-β signaling pathway and EMT in harvested lung tissues. As shown in Figure 7, we first confirmed that metformin decreased expression of α-actin in lung tissues that was induced by bleomycin and/or gefitinib treatment by immunohistochemistry staining and Western blot assay. Next, Western blot analysis showed that Smad2, Smad3, STAT3, AKT and ERK1/2 were strongly phosphorylated in the BLM group and the BLM plus Gef group, while metformin inhibited the activation of those proteins. Finally, we examined EMT phenotype in lung tissues. As shown in Figure 8, very high expression of Vimentin was found in both the BLM group and the BLM plus Gef group, while metformin significantly decreased Vimentin expression; however, the expression of E-cadherin did not show much difference in each group. Taken together, these data show that the therapeutic advantage of metformin was associated with its ability to reverse EMT and inhibit TGF-β signaling *in vivo*.

## DISCUSSION

Administration of EGFR-TKI is first-line therapy in certain NSCLC patients. Although this class of agent is considered relatively safe, the most serious, but comparatively rare adverse reaction is drug-associated interstitial lung disease (ILD). Therefore, it is necessary to find novel strategies to prevent the development of this adverse effect.

### Clinical significance: metformin as a promising new agent to both prevent EGFR-TKI induced pulmonary fibrosis and enhance TKI efficacy

In the present study, we provide compelling evidence that metformin attenuated pulmonary fibrosis from the following aspects. First, we used TGF-β to stimulate pulmonary fibrosis in lung fibroblast HFL-1 cells, since TGF-β is a well-established inducer of fibrosis. TGF-β treatment induced fibrosis in HFL-1 cells, and addition of metformin effectively decreased the expression of those fibrotic markers. Thus, metformin can protect against TGF-β-induced pulmonary fibrosis. Next, we set up a cellular model in which fibrosis was induced by conditioned medium from gefitinib-treated PC-9 cells or PC-9GR cells. PC-9GR cells are gefitinib resistant and are derived from parental sensitive PC-9 cells with an EGFR exon 19 deletion. Conditioned medium from gefitinib-treated PC-9 cells or PC-9GR cells mimics the tumor microenviroment of lung cancer patients that receive gefitinib therapy. As expected, conditioned medium from both systems induced high expression of fibrotic markers (i.e., COL1A1 and α-actin), while metformin addition significantly decreased expression of both of those markers. These data confirmed that metformin could protect against gefitinib-induced pulmonary fibrosis *in vitro*. Lastly, we tested the effect of metformin to attenuate TKI-induced pulmonary fibrosis in a rat model of gefitinib-exacerbated pulmonary fibrosis, which was based on bleomycin-induced acute lung injury, and as described earlier by Takushi et al. [25]. Consistent with their reports, oral administration of gefitinib significantly increased the extent of lung injury and enhanced pulmonary fibrosis. Interestingly, the use of metformin decreased the extent of lung injury and lowered the expression levels of the fibrosis markers COL1A1 and α-actin. Taken together, the findings from the current study suggested that metformin might be a promising agent to protect against the side-effects of EGFR-TKI, and may block pulmonary fibrosis.

These results are of clinical significance. Metformin is a safe and cheap oral antidiabetic drug that used for many years. In recent years, a number of retrospective epidemiological studies and pre-clinical studies have supported the use of metformin as an adjuvant in chemotherapy for cancer treatment [26]. Currently, there are a number of clinical trails using metformin as an adjuvant in chemotherapy (https://www.clinicaltrials.gov/ct2/results?term=metformin+AND+cancer&Search=Search). In addition to the potential role of metformin to prevent EGFR-TKI-induced pulmonary fibrosis, it may simultaneously enhance the anti-tumor effect of EGFR-TKI. One previous study showed that metformin in combination with gefitinib significantly enhanced the efficacy of targeted therapy [27]. Also, our group has previously demonstrated that metformin not only enhances the anti-tumor effect of gefitinib in PC-9 established xenografts, but also reversed TKI resistance in TKI-resistant cancer cells and in PC-9GR xenografts [22]. Also, a retrospective study from our group showed that in advanced NSCLC patients with type 2 diabetes, metformin together with EGFR-TKI resulted in longer PFS and OS [28]. Thus, the dual effects of metformin to both enhance TKI cytotoxicity and prevent TKI-induced pulmonary fibrosis makes it an ideal candidate for combinatorial therapy with EGFR-TKIs in NSCLC patients with activated EGFR mutations with the aim of achieving optimal clinical efficacy with minimal side-effect profiles.

### Metformin protects against EGFR-TKI-induced fibrosis by inhibiting TGF-β signaling

Understanding the molecular mechanisms that underpin the ability of metformin to attenuate EGFR-TKI-induced fibrosis is pivotal in the development of this agent as a novel therapeutic approach in NSCLC patients. It has been reported previously, that inhibition of TGF-β alone may inhibit pulmonary fibrosis [29, 30]. Given the pivotal role of TGF-β in inducing fibrosis, in the present study, we tested whether metformin prevented EGFR-TKI-induced fibrosis by inhibiting the TGF-β signaling pathway. Using ELISA, we found that metformin significantly decreased TGF-β levels in EGFR-TKI-treated lung cancer cells. Furthermore, the role of TGF-β in EGFR-TKI-induced pulmonary fibrosis was determined using a TGF-β mAb. Treatment with TGF-β mAb abolished gefitinib-induced fibrosis, suggesting that TGF-β plays an essential role in mediating TKI-induced fibrosis. Given that metformin significantly decreased activation of the TGF-β signaling pathway, and attenuated TGF-β-induced pulmonary fibrosis, we concluded that metformin attenuated EGFR-TKI-induced pulmonary fibrosis by inhibiting the TGF-β signaling pathway.

### Metformin attenuates EGFR-TKI-induced pulmonary fibrosis by EMT reversal

EMT is a dynamic cellular process that allows polarized, immotile epithelial cells to convert into motile mesenchymal cells [31]. In addition to the essential role that EMT plays in tissue remodeling and tumor metastasis, emerging *in vivo* evidence also elucidates EMT as an important source of myofibroblasts in progressive pulmonary, renal and hepatic fibrosis [16, 32, 33]. Here, we observed that metformin treatment diminished the occurrence of the EMT phenotype in parenchymal alveolar areas following BLM and/or Gef therapy, which suggested that the anti-fibrotic effects of metformin is at least partly due to its interference with TGF-β1-induced EMT.

### Limitations of the current study

The current study have several limitations. First, for *in vivo* experiments, we used a rat model of gefitinib-exacerbated pulmonary fibrosis based on bleomycin-induced acute lung injury, which was reported by Takushi et al [25]. Of note, there may be differences in pathophysiological mechanisms of pulmonary fibrosis induced by bleomycin and gefitinib. The best animal model should be using gefitinib alone to induce pulmonary fibrosis. However, the incidence of EGFR-TKI-associated ILD happens at a very low rate, which means that hundreds of animals will be needed to get a single experiment performed. So we chose the model reported by Takushi et al, yet the potential difference of pulmonary fibrosis induced by bleomycin and gefitinib should be noted. Second, the complete toxic profiles of taking metformin and EGFR-TKIs together was not studied throughly in the current study. In *in vivo* experiments, light to moderate diarrhea was noticed in BLM + MET group and BLM + GEF + MET group. The weight of each animal had been recorded and results showed that there was no significant difference between each group. So in the current study, we did not notice obvious adverse reactions in *in vivo* experiments. However, detailed toxic profiles of this drug combination should be studied before clinical administration of these drugs to patients.

In summary, we have shown that metformin attenuated EGFR-TKI-induced pulmonary fibrosis both *in vitro* and *in vivo* by inhibiting the TGF-β signaling pathway. Given that metformin is safe, cheap and widely used to treat individuals with type 2 diabetes, obesity, and polycystic ovarian syndrome, we propose that metformin has potentially marked clinical utility in the future, since ILD events are an important consideration in the development of EGFR-TKIs. Metformin can also be used in combination with EGFR-TKIs in selected NSCLC patients to increase the efficacy of TKIs and in an attempt to prevent the potential side-effect of pulmonary fibrosis.

## MATERIALS AND METHODS

### Cell-lines and reagents

Gefitinib (Iressa) was purchased from Tocris Bioscience and prepared in dimethyl sulfoxide (DMSO) to obtain a stock solution of 10 mM. Metformin (Sigma) was dissolved in deionized water and stored at −20°C. Dorsomorphin (an AMPK inhibitor) was got from Selleck. Gefitinib-sensitive PC-9 cells and gefitinib-resistant PC-9GR cells were a kind gift from Professor Jun Xu and Dr. Ming Liu from Guangzhou Medical University, China. The HFL1 (human fetal lung fibroblast) cell-line was obtained from the American Type Culture Collection (Manassas, VA, USA). HFL-1 cells were cultured in F-12K medium (Gibco) and all other cells were cultured in Roswell Park Memorial Institute 1640 medium (RPMI-1640, HyClone) with Earle's salts, and supplemented with 10% fetal bovine serum (FBS, Gibco), 2 mM L-glutamine solution (Gibco), 100 U/ml penicillin (HyClone) and 100 μg/ml streptomycin sulfate (HyClone) at 37°C, with 5% CO_2_ in air and 90% humidity.

### Cell growth, and apoptosis assays

The cytotoxic effects of TGF-β plus metformin were determined by the MTT dye reduction assay. A total of 2000 cells were plated in 100 μl culture medium in 96-well microtiter plates. After 24-h incubation, 10 ng/ml TGF-β, and/or 5 mM metformin were added to each well as indicated, and cells were further cultured for 48 h. Then, 10 μl of 5 mg/ml MTT reagent (Sigma) in 100 μl culture medium was added to each well. After 4 h, medium was removed and 150 μl of DMSO was added to each well to dissolve the formazan crystals. Absorbance was measured at a wavelength of 490 nm using a ThermoFisher Spectrophotometer1510 (Molecular Devices, Inc.). Cell viability was determined by dividing the absorbance values of treated cells to that of untreated cells. Experiments were conducted in triplicates.

Cell proliferation was also assessed by the Ki67 incorporation assay using a Ki67 labeling and detection kit (Sigma). Briefly, cells were treated with metformin (5 mM) or 10 ng/ml TGF-β, or both, for 48 h, and then incubated for 6 h with Ki67 (1:200 dilution) and fixed. Cell nuclei were counterstained with 4′, 6-diamidino-2-phenylindole (DAPI) and then viewed with a live cell station (Delta Vision, API). At least 500 cells from three independent experiments were counted. Data were expressed as the mean value of the percentage of positive cells ± SEM.

Flow cytometric analysis was adopted to detect apoptosis by examining altered plasma membrane phospholipid packing by the lipophilic dye Annexin V. Briefly, cells were treated with 10 ng/ml TGF-β and/or 5 mM metformin for 48 h, harvested by trypsin, washed twice with PBS, and then resuspended at a density of 1 × 10^*7*^ cells/mL. Thereafter, 5 μL of Annexin V-FITC and 5 μL of propidium iodide (PI) were added to 100 μL of the cell suspension and incubated for 30 min at room temperature and in the dark. Next, labeled cells were processed by flow cytometry. All early apoptotic cells (i.e., Annexin V-positive, PI-negative), necrotic/late apoptotic cells (i.e., double positive), and living cells (i.e., double negative) were detected by using a Cytomics FC 500 (Beckman Coulter, Miami, FL, USA).

### Western blot assay

Cells grown and treated as indicated were collected and total protein was extracted. The following primary antibodies were used: rabbit monoclonal anti-α-actin (Abcam), monoclonal anti-COL1A1 (Millipore), rabbit monoclonal anti-phosphorylated SMAD2, rabbit monoclonal anti-phosphorylated SMAD3, rabbit monoclonal anti- SMAD2/3, rabbit monoclonal anti-ERK1/2, or rabbit monoclonal anti-phosphorylated ERK1/2, rabbit monoclonal anti-Akt, rabbit monoclonal anti-phosphorylated Akt, rabbit monoclonal anti-STAT3, or rabbit monoclonal anti-phosphorylated STAT3 (all from Cell Signaling Technology, Inc.). Horseradish peroxidase-conjugated goat-anti-rabbit antibody (Thermo Scientific) was used as a secondary antibody. The control for equal protein loading was assessed with an anti-β-actin antibody (Cell Signaling Technology, Inc.).

### Animal experiments

For animal experiments, male SD rats with an average weight of approximately 200 g (Laboratory Animal Center of Third Military Medical University, Chongqing, China) were maintained under chloral hydrate anesthesia (500 mg/kg) and given one intratracheal injection of bleomycin (5 mg/kg) to induce fibrosis. Gefitinib (200 mg/kg) was dissolved in 1% methylcellulose and administered orally once per day for three days before animals received a single intratracheal administration of bleomycin; the administration of gefitinib and metformin (300 mg/kg) was then continued, once every two days, for the following 21 days. The animals were kept in individual ventilated cages in compliance with institutional guidelines. All animal protocols were approved by the local Ethics Committee of the Third Military Medical University. After 21 days, rats were sacrificed, and the lungs were harvested, fixed in 4% paraformaldehyde and embedded in paraffin.

### Immunohistochemistry

For immunofluorescence, cells were washed with PBS and fixed in 4% paraformaldehyde at room temperature for 30 min. Lung tissues fixed in 4% paraformaldehyde for 48 h were embedded in paraffin and cut into 4-μm thick sections. Non-specific binding was blocked using 10% normal goat serum (Sigma). Cells or lung sections were incubated with the following primary antibodies after being diluted in PBS with 1% bovine serum albumin at 4°C overnight: mouse monoclonal anti E-cadherin (Cell Signaling Technology, Inc.) and rabbit monoclonal anti-Vimentin (Cell Signaling Technology, Inc.). Then, cells or tumor sections were washed twice with PBS and incubated with secondary antibodies at 37°C for 30 min as follows: FITC-conjugated goat-anti-rabbit IgG (Abcam) or TRITC-conjugated goat-anti-mouse IgG (Sigma). The slides were mounted in mounting medium with 4′, 6-diamidino-2-phenylindole (DAPI; Vector Laboratories) and viewed with a live cell station (Delta Vision, API).

### Hydroxyproline determination

Hydroxyproline content was determined as previously described [34]. Briefly, the right lung was removed and homogenized in 0.5 ml of 5% TCA. After centrifugation, pellets were hydrolyzed in 0.5 ml of 10 N HCl for 16 h at 110°C. Each sample was incubated for 20 min at room temperature after the addition of 0.5 ml of 1.4% (w/v) chloramine-T solution and then incubated at 65°C for 10 min after the addition of 0.5 ml of Ehrlich's reagent (1 M DMBA, 70% (v/v) isopropanol and 30% (v/v) perchloric acid). Absorbance was measured at a wavelength of 550 nm and the amount of hydroxyproline was determined.

### Statistical analysis

All data are presented as mean ± standard error of the mean (SEM). Statistical analyses were carried out using the unpaired, two-tailed Student's *t* test and statistical significance was assumed at an alpha value of *p* < 0.05.
